# Increase in negative charge of ^68^Ga/chelator complex reduces unspecific hepatic uptake but does not improve imaging properties of HER3-targeting affibody molecules

**DOI:** 10.1038/s41598-019-54149-3

**Published:** 2019-11-27

**Authors:** Sara S. Rinne, Charles Dahlsson Leitao, Joshua Gentry, Bogdan Mitran, Ayman Abouzayed, Vladimir Tolmachev, Stefan Ståhl, John Löfblom, Anna Orlova

**Affiliations:** 1 Department of Medicinal Chemistry, Uppsala University, 751 23 Uppsala, Sweden; 2 Department of Protein Science, School of Engineering Sciences in Chemistry, Biotechnology and Health, KTH Royal Institute of Technology, 106 91 Stockholm, Sweden; 3 Department of Immunology, Genetics and Pathology, Uppsala University, 751 85, Uppsala, Sweden; 4 Science for Life Laboratory, Uppsala University, 751 23 Uppsala, Sweden

**Keywords:** Cancer imaging, Diagnostics

## Abstract

Upregulation of the human epidermal growth factor receptor type 3 (HER3) is a common mechanism to bypass HER-targeted cancer therapy. Affibody-based molecular imaging has the potential for detecting and monitoring HER3 expression during treatment. In this study, we compared the imaging properties of newly generated ^68^Ga-labeled anti-HER3 affibody molecules (HE)_3_-Z_HER3_-DOTA and (HE)_3_-Z_HER3_-DOTAGA with previously reported [^68^Ga]Ga-(HE)_3_-Z_HER3_-NODAGA. We hypothesized that increasing the negative charge of the gallium-68/chelator complex would reduce hepatic uptake, which could lead to improved contrast of anti-HER3 affibody-based PET-imaging of HER3 expression. (HE)_3_-Z_HER3_-X (X = DOTA, DOTAGA) were produced and labeled with gallium-68. Binding of the new conjugates was specific in HER3 expressing BxPC-3 and DU145 human cancer cells. Biodistribution and *in vivo* specificity was studied in BxPC-3 xenograft bearing Balb/c nu/nu mice 3 h pi. DOTA- and DOTAGA-containing conjugates had significantly higher concentration in blood than [^68^Ga]Ga-(HE)_3_-Z_HER3_-NODAGA. Presence of the negatively charged ^68^Ga-DOTAGA complex reduced the unspecific hepatic uptake, but did not improve overall biodistribution of the conjugate. [^68^Ga]Ga-(HE)_3_-Z_HER3_-DOTAGA and [^68^Ga]Ga-(HE)_3_-Z_HER3_-NODAGA had similar tumor-to-liver ratios, but [^68^Ga]Ga-(HE)_3_-Z_HER3_-NODAGA had the highest tumor uptake and tumor-to-blood ratio among the tested conjugates. In conclusion, [^68^Ga]Ga-(HE)_3_-Z_HER3_-NODAGA remains the favorable variant for PET imaging of HER3 expression.

## Introduction

The human epidermal growth factor receptor type 3 (HER3) is recognized as an important target in anti-cancer therapy^1,2^. HER3 is a member of the HER-family with a regulatory function and the ability to activate the PI3K/AKT downstream signaling pathway^3,4^. However, its impaired intrinsic tyrosine kinase activity requires the formation of heterodimeric complexes with other HER-receptors for downstream signaling^1^. Its involvement in different cancer subtypes e.g. breast, gastric and prostate cancers has been documented^4–6^. Cancer-related overexpression of HER3 is often seen as a poor prognostic factor^7–9^ and is strongly linked to resistance against targeted therapy^10–13^. The upregulation of HER3 can activate alternative signaling routes, thus circumventing the pathways inhibited by the therapeutic agent^12,14^. Changes in expression of tyrosine kinase receptors such as HER3 can occur already few hours after treatment administration^15^. Co-expression of HER3 is therefore critical information for efficient patient management and blocking of HER3-mediated signaling might be a strategy to circumvent therapy resistance^11^. HER3-targeted therapies, including mono- and bivalent antibodies, are in different stages of clinical trials^2^.

For effective treatment, it is essential to reliably detect upregulation and monitor the status of HER3 expression. Radionuclide-based molecular imaging with positron emission tomography (PET) and single-photon emission tomography (SPECT) is a promising approach for non-invasive and repeatable assessment of target expression. The possibility for repetitive scans and in the case of PET also quantification is of particular interest for HER3 expression because of its dynamic oncogenic expression.

High contrast imaging of HER3 expression in cancer lesions is generally challenging. Overexpression in the malignant tissue is typically below 50.000 receptors/cells^16^ and healthy organs, such as liver, endogenously express HER3. Several studies have reported monoclonal antibodies labeled with ^89^Zr for imaging of HER3 expression and monitoring of HER3 status during treatment^17–19^. However, antibodies clear slowly from blood and therefore typically only provide suitable imaging contrast days after administration, which also prevents serial imaging within short time intervals. Furthermore, the enhanced permeability and retention effect, and hepatobiliary excretion limit the contrast in HER3 positive lesions and therefore clinical application^17^. The use of smaller imaging agents with faster kinetics and better tumor penetration, such as F(ab’)_2_ fragments, nanobodies, peptides or engineered scaffold proteins might be preferable^15,20,21^.

Affibody molecules are a class of engineered scaffold proteins with small size (7–8 kDa), fast kinetics, extravasation and clearance, and good tumor penetration^22,23^. This makes them suitable candidates for molecular imaging. For example, ^68^Ga-labeled HER2 (Human Epidermal Growth Factor Receptor 2) detecting affibody molecules have shown promising results in clinical trials^24^. Capable of visualizing both high and low HER2 expressing lesions, they could successfully identify the HER2-status of breast cancer patients^24^.

We have earlier reported on HER3-targeting affibody molecules radiolabeled with both PET and SPECT-isotopes as a promising alternative for imaging of HER3 expression^25–28^. Radiolabeled affibody molecules for imaging could be a suitable theranostic companion for future antibody or affibody based therapeutic agents against HER3^29–31^. So far, the results are encouraging, but HER3 expression in healthy organs together with comparably low expression in tumors still pose challenges in achieving high imaging contrast. Generally, the imaging contrast could be improved by increasing tumor uptake or by decreasing uptake in healthy tissue (or both).

Hepatic metastases are common in many cancers and our recent efforts have focused on improving the imaging contrast by reducing the hepatic uptake. Liver uptake is mediated by the natural expression of HER3 but also partially related to unspecific or “off-target”-interactions which can be influenced by local charge of the targeting molecule and its hydrophilicity/lipophilicity^32–35^. We have previously demonstrated that co-injection of unlabeled trivalent affibody can block endogenous HER3 receptors in the liver to a greater extent than in the tumor, which consequently dramatically increased the tumor-to-liver contrast^36^.

Another approach is to focus on the molecular design of the affibody molecules to reduce unspecific uptake. Differences in structure of the metal/chelator-complex, surface exposure of functional groups and local distribution of charge, have shown to influence blood clearance, renal, hepatic and tumor uptake of anti-HER2 affibody molecules^37–39^. Particularly, presence of negative charged complexes can result in reduction of non-specific hepatic uptake. We also demonstrated that this is applicable to anti-HER3 affibody molecules^28,40,41^. Comparison of different indium-111/chelator complexes (^111^In-NOTA, ^111^In-NODAGA, ^111^In-DOTA, ^111^In-DOTAGA) conjugated to the C-terminus of Z_HER3_ showed that exchanging the positively charged ^111^In-NOTA-complex with a negatively charged ^111^In-DOTAGA resulted in a two-fold reduction in hepatic uptake and clearly improved the tumor-to-liver contrast^41^. Recently, we investigated the influence of a hydrophilic N-terminal (HE)_3_-tag on the ^68^Ga-labeled anti-HER3 affibody molecules Z_HER3_^40^. The study included a head-to-head comparison of (HE)_3_-Z_HER3_ with Z_HER3_ labeled with ^68^Ga via a NOTA (1,4,7-triazacyclononane-N,N0,N0 0-triacetic acid) or NODAGA (1-(1,3-carboxypropyl)-4,7-carboxymethyl-1,4,7-triazacyclononane) chelator. Presence of the hydrophilic (HE)_3_-tag increased the clearance rate from blood and reduced activity uptake in normal tissue, including liver, which resulted in increased tumor-to-liver ratios. In the same study, we found that NODAGA, which is neutrally charged when loaded with trivalent metals, was favorable compared to positively charged NOTA.

Gallium-68 is a clinically available PET isotope, not only allowing detection but also quantification of HER3 expression^27^. Based on our experience with indium-111^41^, we hypothesized that a further increase in negative charge of the ^68^Ga-chelator complex could potentially improve PET-imaging contrast and clinical utility of the (HE)_3_-containing tracer. In the present study, we therefore compared ^68^Ga-labeled DOTA- (1,4,7,10-Tetraazacyclododecane-1,4,7,10-tetraacetic acid) and DOTAGA-conjugated (1,4,7,10-tetraazacyclododececane,1-(glutaric acid)-4,7,10-triacetic acid) variants of (HE)_3_-Z_HER3_ with [^68^Ga]Ga-(HE)_3_-Z_HER3_-NODAGA with the aim to further improve image contrast.

## Results

### Production

The (HE)_3_-tagged HER3-binding affibody, (HE)_3_-Z_HER3_, was produced in *E. coli* and purified by IMAC, followed by coupling to maleimide derivatives of DOTA and DOTAGA. The purity, determined with RP-HPLC, exceeded 95% for all conjugates (Fig. S1). The experimental molecular mass of each conjugate was in perfect agreement with the theoretical mass (Fig. S2). Notably, the mass determination revealed non-processed N-terminal methionine for all conjugates, due to the presence of the (HE)_3_-tag at the N-terminus. The alpha-helical content, thermal stability, refolding of the conjugates and melting temperatures were investigated by circular dichroism spectroscopy (Fig. S3, Table S1). Binding affinities were measured with surface plasmon resonance (SPR) analysis and K_D_ values are presented in Table 1 as the average from duplicate injections. K_D_ values refer to the monovalent affinity for human HER3 according to a Langmuir 1:1 model. Sensorgrams with fitted curves are shown in Fig. S4.

**Table 1:** Experimental molecular masses (Mw) and equilibrium dissociation constants (K_D_) of the conjugates. K_D_ values are presented as the average from duplicate injections.

	Mw (Da)	K_D_ (pM, mean ± SD)
(HE)_3_-Z_HER3_-NODAGA*	8221.2 (8221.1)*	38 ± 10*
(HE)_3_-Z_HER3_-DOTA	8250.3 (8250.2)	24 ± 1.3
(HE)_3_-Z_HER3_-DOTAGA	8322.3 (8322.2)	39 ± 3.3

The theoretical molecular mass is in parenthesis. *Data for [^68^Ga]Ga-(HE)_3_-Z_HER3_-NODAGA was previously reported by^40^.

A more detailed description of the results of the production and characterization of the affibody molecules is included in the Supplementary Materials. Production and characterization of (HE)_3_-Z_HER3_-NODAGA was previously described^40^, but is also provided in Supplementary Materials for comparison.

### Labeling and stabilty

Labeling yield and stability data for all conjugates are presented in Table 2. [^68^Ga]Ga(HE)_3_-Z_HER3_-DOTA and [^68^Ga]Ga-(HE)_3_-Z_HER3_-DOTAGA were labeled with gallium-68 in sodium acetate buffer (1.25 M, pH 3.6). Radiochemical yields after incubation at 85 °C for 15 minutes were 53 ± 30% and 81 ± 16% for (HE)_3_-Z_HER3_-DOTA and (HE)_3_-Z_HER3_-DOTAGA, determined with instant thin layer chromatography (ITLC). Intermediate EDTA (Ethylenediaminetetraacetic acid) challenge removed loosely bound gallium-68 from the affibody molecules and resulted in 7 ± 2% release of affibody-associated activity for [^68^Ga]Ga-(HE)_3_-Z_HER3_-DOTA and 11 ± 6% for [^68^Ga]Ga-(HE)_3_-Z_HER3_-DOTAGA. After purification with NAP5-size exclusion columns, purity exceeded 99% for both conjugates.

**Table 2:** Labeling and stability of [^68^Ga]Ga-(HE)_3_-Z_HER3_-DOTA, [^68^Ga]Ga-(HE)_3_-Z_HER3_-DOTAGA, [^68^Ga]Ga-(HE)_3_-Z_HER3_-NODAGA. Stability of the radiolabeled complexes is expressed as % release (n = 6).

Analysis	[^68^Ga]Ga-(HE)_3_-Z_HER3_-NODAGA (n = 3)	[^68^Ga]Ga-(HE)_3_-Z_HER3_-DOTA (n = 3)	[^68^Ga]Ga-(HE)_3_-Z_HER3_-DOTAGA (n = 5)
Radiochemical yield (%), % Release of ^68^Ga in EDTA challenge (1000× EDTA, 10 min, 85 °C)	97 ± 2*	53 ± 30	81 ± 16
		7 ± 2	11 ± 6
Purity after NAP5 size exclusion (%)	<99*	99.7 ± 0.5	99.8 ± 0.2
% release in PBS, 1 hour	0.03 ± 0.05*	0.4 ± 0.2	0.3 ± 0.4
% release in human serum, 1 hour 37 °C	0.23 ± 0.05*	3.7 ± 0.7	1 ± 1

*Data for [^68^Ga]Ga-(HE)_3_-Z_HER3_-NODAGA was previously reported by^40^.

Following purification, both conjugates showed no major release of the radiolabel when incubated in phosphate-buffered saline (PBS). After incubation in human serum [^68^Ga]Ga-(HE)_3_-Z_HER3_-DOTA showed a somewhat higher fraction of free gallium than [^68^Ga]Ga-(HE)_3_-Z_HER3_-DOTAGA. Labeling of (HE)_3_-Z_HER3_-NODAGA resulted in almost quantitative yields and purity > 99% matching previously reported results^40^.

### *In vitro* characterization

HER3 expressing human cancer cell lines BxPC-3 (pancreatic carcinoma) and DU145 (prostate cancer) were used for *in vitro* characterization of [^68^Ga]Ga-(HE)_3_-Z_HER3_-DOTA and [^68^Ga]Ga-(HE)_3_-Z_HER3_-DOTAGA. [^68^Ga]Ga-(HE)_3_-Z_HER3_-NODAGA was previously characterized^40^.

**Figure 1:**
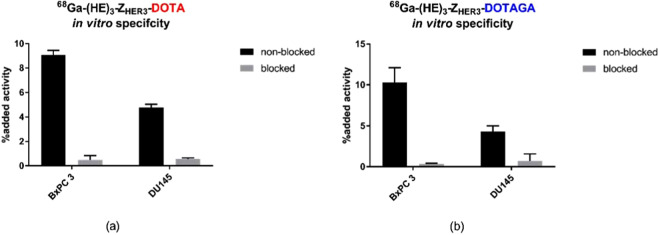
*In vitro* specificity test of **(a)** [^68^Ga]Ga-(HE)_3_-Z_HER3_-DOTA and **(b)** [^68^Ga]Ga-(HE)_3_-Z_HER3_-DOTAGA on BxPC-3 and DU145 cells (n = 3 per datapoint). In the blocked groups, HER3 receptors were pre-saturated with 50 nM of unlabeled Z_HER3_. Binding specificity of [^68^Ga]Ga-(HE)_3_-Z_HER3_-NODAGA was previously demonstrated^40^.

The results of the binding specificity experiment are illustrated in Fig. 1. Cells were incubated with 0.1 nM of [^68^Ga]Ga-(HE)_3_-Z_HER3_-DOTA or [^68^Ga]Ga-(HE)_3_-Z_HER3_-DOTAGA for 1 hour. In the blocked groups, HER3 receptors were pre-saturated by addition of 50 nM unlabeled Z_HER3_, resulting in a significant decrease of activity uptake. Thus, binding of [^68^Ga]Ga-(HE)_3_-Z_HER3_-DOTA and [^68^Ga]Ga-(HE)_3_-Z_HER3_-DOTAGA was HER3-mediated. Overall uptake of the conjugates in DU145 cells was lower than in BxPC-3 cells.

**Figure 2:**
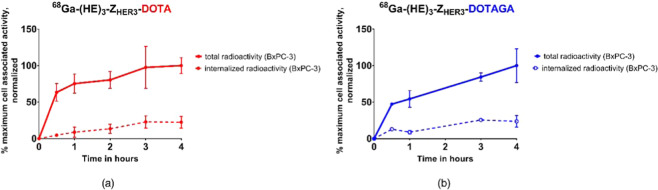
Cellular processing on BxPC-3. Cells were continuously incubated with 0.1 nM of **(a)** [^68^Ga]Ga-(HE)_3_-Z_HER3_-DOTA or **(b)** [^68^Ga]Ga-(HE)_3_-Z_HER3_-DOTAGA for 4 hours. Experiments were performed on both cell lines in parallel using the same stock solution of the radiolabeled affibody molecules (n = 3 per datapoint). Cellular processing of [^68^Ga]Ga-(HE)_3_-Z_HER3_-NODAGA was previously described^40^.

Cellular processing was studied by continuously incubating BxPC-3 and DU145 cells with 0.1 nM of the radiolabeled conjugates for up to 4 hours. At preselected time points, the membrane bound activity and internalized fractions were collected for BxPC-3 cells. For DU145 cells, only the total cell associated activity was studied, because of low signal due to the low level of HER3 expression. Figure 2 shows the uptake pattern of the activity, normalized to the maximum cell associated activity in BxPC-3 cells. Data for DU145 cells can be found in the Supplementary Material (Fig. S5). The binding of both conjugates to the cells was quick and increased in BxPC-3 cells over time. After 4 h the fraction of internalized activity was 23 ± 8% for [^68^Ga]Ga-(HE)_3_-Z_HER3_-DOTA and 24 ± 8% for [^68^Ga]Ga-(HE)_3_-Z_HER3_-DOTAGA. Uptake in DU145 cells was lower compared to uptake in BxPC-3 cells. The conjugates also associated quickly, but uptake did not increase over time.

### *In vivo* experiments

For *in vivo* experiments, female Balb/c nu/nu mice bearing BxPC-3 xenografts were injected with 2 µg (0.7 MBq) [^68^Ga]Ga-(HE)_3_-Z_HER3_-NODAGA, [^68^Ga]Ga-(HE)_3_-Z_HER3_-DOTA or [^68^Ga]Ga-(HE)_3_-Z_HER3_-DOTAGA. Tumors and tissue samples were collected 3 h pi. For *in vivo* specificity test, the amount of injected protein was adjusted to 70 µg using unlabeled anti-HER3 affibody.

**Figure 3:**
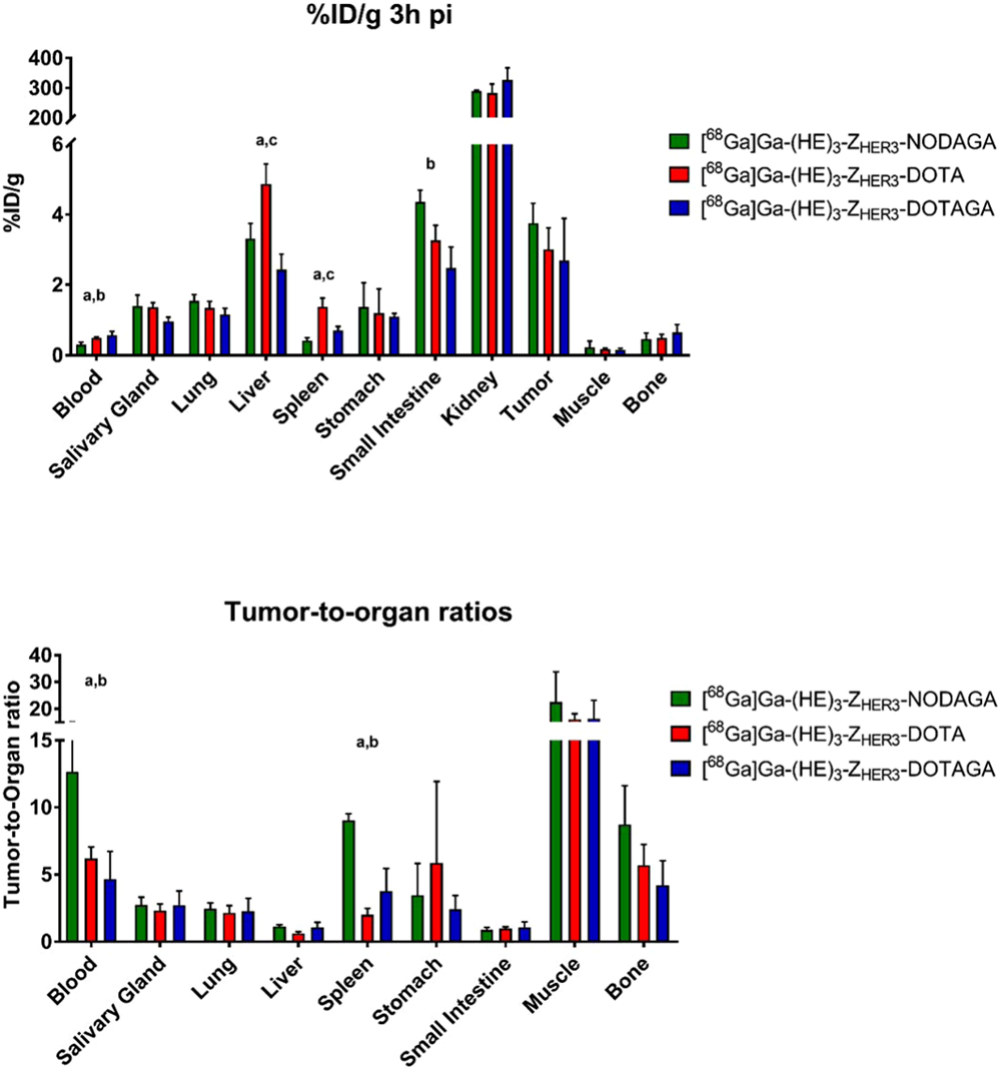
*In vivo* biodistribution 3 h pi: (**Top**) activity concentrations as %ID/g and **(Bottom)** tumor-to-organ ratios. Female Balb/c nu/nu mice were injected with 2 µg of [^68^Ga]Ga-(HE)_3_-Z_HER3_-X (n = 3–4 animals per group). Differences were statistically significant (p < 0.05) between a) [^68^Ga]Ga-(HE)_3_-NODAGA and [^68^Ga]Ga-(HE)_3_-DOTA, b) [^68^Ga]Ga-(HE)_3_-NODAGA and [^68^Ga]Ga-(HE)_3_-DOTAGA, c) [^68^Ga]Ga-(HE)_3_-DOTA and [^68^Ga]Ga-(HE)_3_-DOTAGA.

All conjugates bound to the tumors without significant differences (Fig. 3 (top)). Tumor uptake was in the range of 2.7 to 3.7%ID/g. Characteristic for affibody molecules, activity cleared quickly from the blood (concentration below 0.6%ID/g, 3 h pi). Significantly lower activity concentration in blood was observed for [^68^Ga]Ga-(HE)_3_-Z_HER3_-NODAGA. All conjugates had elevated uptake in organs with mErbB3 expression, which was expected since Z_HER3_ is crossreactive for the murine orthologue. However, uptake of [^68^Ga]Ga-(HE)_3_-Z_HER3_-DOTAGA tended to be lower than the other variants in HER3 expressing organs, especially the liver, salivary glands and intestines. [^68^Ga]Ga-(HE)_3_-Z_HER3_-DOTA had clearly the highest uptake in the liver among the tested conjugates. Hepatic uptake was 4.9 ± 0.6%ID/g for [^68^Ga]Ga-(HE)_3_-Z_HER3_-DOTA while 3.3 ± 0.4%ID/g for [^68^Ga]Ga-(HE)_3_-Z_HER3_-NODAGA and 2.4 ± 0.4%ID/g for [^68^Ga]Ga-(HE)_3_-Z_HER3_-DOTAGA. [^68^Ga]Ga-(HE)_3_-Z_HER3_-DOTA uptake in the spleen was also 2–3 fold higher than for the NODAGA- and DOTAGA-conjugated variants.

Injection of excess amount of unlabeled HER3-targeting affibody molecules resulted in significant decrease of uptake in mErbB3 expressing organs and in the tumors, confirming HER3-mediated uptake of the molecules (Table 3). Still, even with co-injection of excess amount of protein, activity concentration in the liver was more than 2-fold higher for [^68^Ga]Ga-(HE)_3_-Z_HER3_-DOTA than for [^68^Ga]Ga-(HE)_3_-Z_HER3_-DOTAGA.

**Table 3:** *In vivo* specificity of [^68^Ga]Ga-(HE)_3_-Z_HER3_-DOTA, [^68^Ga]Ga-(HE)_3_-Z_HER3_-DOTAGA (n = 3–4 animals per group).

Organ	[^68^Ga]Ga-(HE)_3_-Z_HER3_-DOTA		[^68^Ga]Ga-(HE)_3_-Z_HER3_-DOTAGA	
	2 µg	70 µg	2 µg	70 µg
Blood	0.49 ± 0.03^*^	0.36 ± 0.02	0.6 ± 0.1	0.40 ± 0.05
Salivary gland	1.4 ± 0.1^*^	0.35 ± 0.04	1.0 ± 0.1^*^	0.33 ± 0.03
Lung	1.3 ± 0.2^*^	0.45 ± 0.08	1.2 ± 0.2^*^	0.39 ± 0.03
Liver	4.9 ± 0.6^*^	2.73 ± 0.10	2.4 ± 0.4^*^	1.0 ± 0.2
Spleen	1.4 ± 0.3	1.0 ± 0.1	0.7 ± 0.1	0.5 ± 0.1
Stomach	1.2 ± 0.7	0.26 ± 0.02	1.10 ± 0.09^*^	0.3 ± 0.1
Small intestine	3.3 ± 0.4^*^	0.16 ± 0.02	2.5 ± 0.6^*^	0.37 ± 0.08
Kidney	282 ± 31^*^	347 ± 26	326 ± 41	297 ± 27
Tumor	3.0 ± 0.6^*^	0.88 ± 0.09	3 ± 1^*^	0.8 ± 0.2
Muscle	0.17 ± 0.03	0.15 ± 0.04	0.16 ± 0.04	0.13 ± 0.02
Bone	0.5 ± 0.1	0.4 ± 0.2	0.6 ± 0.2	0.41 ± 0.02
GI (%ID)	5.1 ± 0.6^*^	0.5 ± 0.08	3.1 ± 0.4^*^	0.6 ± 0.2
Carcass (%ID)	8 ± 1^*^	2 ± 2	7.1 ± 0.9^*^	3.2 ± 0.5

Specificity of [^68^Ga]Ga-(HE)_3_-Z_HER3_-NODAGA was previously confirmed^40^. *significant difference (p < 0.05) between 2 µg and 70 µg.

Tumor-to-organ ratios are illustrated in Fig. 3 (bottom). Tumor-to-blood ratio of [^68^Ga]Ga-(HE)_3_-Z_HER3_-NODAGA was the highest, being 2-fold higher than [^68^Ga]Ga-(HE)_3_-Z_HER3_-DOTA and 3-fold higher than [^68^Ga]Ga-(HE)_3_-Z_HER3_-DOTAGA. There were no significant differences in tumor-to-lung, -salivary or -small intestine ratios between the conjugates. Tumor-to-liver ratios of [^68^Ga]Ga-(HE)_3_-Z_HER3_-NODAGA and [^68^Ga]Ga-(HE)_3_-Z_HER3_-DOTAGA exceeded 1 and were almost 2-fold higher than for [^68^Ga]Ga-(HE)_3_-Z_HER3_-DOTA.

## Discussion

**Figure 4:**
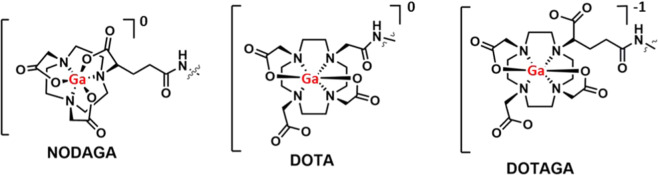
Schematic structures of gallium complexes with NODAGA, DOTA and DOTAGA chelators.

Oncogenic expression of HER3 is dynamic, heterogeneous and a common cause for therapy resistance^11^. Radionuclide-based molecular imaging of HER3 expression could be valuable in evaluation of the status of HER3 expression in cancer patients, in pre- and post-treatment analysis as well as monitoring of treatment progress. Radiolabeled HER3-targeting affibody molecules can visualize and discriminate between different levels of HER3 expression in pre-clinical models^26,27^. We have previously shown that an increase in hydrophilicity using a N-terminal (HE)_3_-tag improves the biodistribution and tumor-to-liver contrast of [^68^Ga]Ga-(HE)_3_-Z_HER3_. Furthermore, we found that, thus far, [^68^Ga]Ga-(HE)_3_-Z_HER3_-NODAGA was the favorable Z_HER3_-variant for PET-imaging with gallium-68^40^. In the present study, our aim was to investigate whether the C-terminal conjugation of tetraaza-chelators DOTA and DOTAGA for labeling of (HE)_3_-Z_HER3_ with gallium-68 would further improve the imaging properties. We therefore compared the newly produced Z_HER3_-variants [^68^Ga]Ga-(HE)_3_-Z_HER3_-DOTA and [^68^Ga]Ga-(HE)_3_-Z_HER3_-DOTAGA with the previously selected [^68^Ga]Ga-(HE)_3_-Z_HER3_-NODAGA (Fig. 4).

All conjugates could be labeled with gallium-68 (Table 2). Average labeling yields of [^68^Ga]Ga-(HE)_3_-Z_HER3_-DOTA and -DOTAGA were lower than previously reported values for [^68^Ga]Ga-(HE)_3_-Z_HER3_-NODAGA^40^. It is possible that the chelators influenced the conformation of the protein making the chelator less accessible or created additional weaker chelating pockets. Therefore, an intermediate EDTA-challenge was included in the labeling protocol for [^68^Ga]Ga-(HE)_3_-Z_HER3_-DOTA and [^68^Ga]Ga-(HE)_3_-Z_HER3_-DOTAGA, to remove weakly bound gallium-68 from these pockets. A similar approach was previously reported by^42^ when an intermediate cysteine challenge was introduced for the labeling of anti-EGFR affibody molecules with ^99m^Tc. In our case, the EDTA challenge slightly reduced the fraction of affibody-associated activity, but resulted in high stability of the purified products, supporting the hypothesis of existing weak binding pockets. Nevertheless, incubated in human serum, [^68^Ga]Ga-(HE)_3_-Z_HER3_-DOTA showed a somewhat higher release of gallium-68 than [^68^Ga]Ga-(HE)_3_-Z_HER3_-DOTAGA and [^68^Ga]Ga-(HE)_3_-Z_HER3_-NODAGA.

The labeled conjugates bound specifically to HER3 receptors *in vitro* and *in vivo* (Fig. 1, Table 3). In BxPC-3 cells, total uptake and the internalized activity increased with time (Fig. 2). Internalization rates for both conjugates were higher compared with our previously reported rates for [^68^Ga]Ga-(HE)_3_-Z_HER3_-NODAGA^40^. The influence of chelators on internalization properties of anti-HER2 and anti-HER3 affibody molecules has previously been reported^37,41^. Within the HER-family, internalization is dependent on receptor expression level and availability of dimerization partners. It is possible that the composition of the radiometal-chelator complex interferes with the heterodimerization of HER3. Overall, uptake was lower in DU145 cells due to lower receptor density.

The overall biodistribution correlated well with previously published data^27,40^. Typically for affibody molecules in general and HER3-targeting specifically, all conjugates cleared quickly from the blood via the renal pathway and accumulated in organs with endogenous expression of mErbB3. The uptake in the HER3-expressing xenografts was in the range of 3 to 4%ID/g. The different C-terminal compositions did not have a significant effect on the tumor uptake, but influenced the uptake in normal organs such as liver, blood, spleen, and small intestine.

Both tetraaza-conjugated variants showed slightly but significantly higher activity concentration in blood compared to [^68^Ga]Ga-(HE)_3_-Z_HER3_-NODAGA. It is possible that presence of free carboxylic groups in [^68^Ga]Ga-DOTA and [^68^Ga]Ga-DOTAGA complexes can change the interaction with blood proteins. This phenomenon was reported earlier for shorter peptides and affibody molecules^38,39^. The higher activity concentration of [^68^Ga]Ga-(HE)_3_-Z_HER3_-DOTA and [^68^Ga]Ga-(HE)_3_-Z_HER3_-DOTAGA in blood translated into 2–3 fold lower tumor-to-blood ratios than for [^68^Ga]Ga-(HE)_3_-Z_HER3_-NODAGA. [^68^Ga]Ga-(HE)_3_-Z_HER3_-DOTA had three-fold higher uptake in spleen and significantly higher hepatic uptake than [^68^Ga]Ga-(HE)_3_-Z_HER3_-NODAGA and -DOTAGA. Elevated uptake in these organs can indicate lower stability of the radiolabel. Release of ^68^Ga from the chelator could result in trans-chelation to transferrin or the formation of gallium-hydroxide colloids, the latter tend to accumulate in the spleen and to some extent in the liver^33,43^. This *in vivo* finding corroborates with literature data and the observed lower *in vitro* stability of [^68^Ga]Ga-(HE)_3_-Z_HER3_-DOTA^44^.

It was previously observed that increasing the negative charge of the radiometal/chelator complex can alter the biodistribution of HER3-targeting affibody molecules and particularly reduce unspecific uptake in the liver^28,33,40,41^. Our data aligned with this observation. [^68^Ga]Ga-(HE)_3_-Z_HER3_-DOTAGA with a negatively charged ^68^Ga/chelator-complex reduced the activity uptake in liver and small intestine compared to NODAGA-conjugated variant with neutral complex charge. However, the tumor-to-liver-ratio, which is an indicator for imaging contrast, was not significantly higher and the tumor-to-blood ratio was 3-fold lower for the DOTAGA-containing conjugate than for [^68^Ga]Ga-(HE)_3_-Z_HER3_-NODAGA. This may indicate that the variant with the most negative charge of the radiometal/chelator complex is not always the most favorable one overall. This is not concurrent with our findings with indium-111, where the ^111^In-DOTAGA complex with the most negative charge also provided the most favorable biodistribution^41^. Indium and gallium isotopes differ in size, coordination number, and complex geometry with DOTA and its derivative DOTAGA^45^. Because of a smaller ionic radius, gallium-68 tends to prefer triaza-chelators, such as NOTA and NODAGA, whereas indium may prefer tetraaza-ligands^44,46,47^. Hence, conclusions from indium-labeled targeting molecules may not always be directly translatable to gallium-labeled variants.

Comparing the molecules included in this study with other small HER3-targeting molecules, both [^68^Ga]Ga-(HE)_3_-Z_HER3_-DOTA and [^68^Ga]Ga-(HE)_3_-Z_HER3_-DOTAGA showed similar or higher tumor-to-liver ratios and higher tumor-to-blood ratios than a ^89^Zr-labeled nanobody^21^ and ^68^Ga-labeled HER3 peptide^20^. Reported tumor uptake of the ^89^Zr-labeled nanobody in xenografted mice was comparable at 3 h pi, and increased at 24 h pi^21^. However, the higher concentration of the nanobody in blood at 3 h pi compared to (HE)_3_-Z_HER3_ is a drawback for high contrast imaging within this timeframe. The reported uptake of the HER3-targeting peptide did not exceed 1%ID/g in the xenografts^20^.

In conclusion, the imaging properties of radiolabeled affibody molecules are determined by many different parameters. In the present study, we hypothesized that an increase in negative charge of the gallium-68/chelator complex would decrease hepatic uptake and increase the contrast of HER3 PET-imaging. The results demonstrated that the negatively charged ^68^Ga-DOTAGA complex indeed reduced the hepatic uptake, but did not improve the overall imaging properties. We therefore conclude that parameters influencing the imaging properties of affibody molecules should be studied for each molecule/isotope individually and that [^68^Ga]Ga-(HE)_3_-Z_HER3_-NODAGA remains the most promising variant for PET imaging of HER3 expression.

## Materials and Methods

### General

Human cancer cell lines BxPC-3 and DU145 were purchased from American type tissue culture collection (ATTC via LGC Promochem, Borås, Sweden).

Metal contaminants were removed from buffers with Chelex100 Resin (Sigma Aldrich, St.Louis, MO, USA).

ITLC was used to measure the distribution of activity to determine labeling yield and stability of the radiolabeled compounds. For analysis, 1 µl of the sample was added to strips made of silica gel-impregnated glass microfiber chromatography paper (Agilent Technologies, Santa Clara, CA, USA). Citric acid (0.2 M) was used for elution. With this method, free gallium-68 will move to the front of the strips and the radiolabeled affibody will stay at the application point. Distribution of activity was measured in the Cyclone Storage Phosphor System and analyzed with OptiQuant image analysis software (Perkin Elmer, Waltham, MA, USA).

Activity in cells and organs samples was measured with an automated gamma counter with a 3-inch NaI(Tl) detector (1480 Wizard; Wallac Oy, Turku, Finland). Raw data were corrected for decay.

Statistical significance for *in vitro* and *in vivo* specificity was tested with two-tailed, unpaired t-test. Comparison of the different groups in the biodistribution was done with 1-way ANOVA and post-hoc t-test corrected for multiple comparisons with the Bonferroni method.

### Production, conjugation and purification

Affibody molecules (HE)_3_-Z_HER3:08698_-DOTA and (HE)_3_-Z_HER3:08698_-DOTAGA were produced, purified and characterized according to previously described methods^40^.

Briefly, the HER3-binding affibody (HE)_3_-Z_HER3:08698_ (further denoted as (HE)_3_-Z_HER3_) was produced in BL21*(DE3) *E. coli* (Thermo Fisher Scientific) in an overnight culture at 25 °C after induced expression with 100 μM isopropyl -D-1-thiogalactopyranoside at an OD_600_ of 0.8.

Cells were lysed with French press and the supernatant was heated to 90 °C for 10 min followed by incubation on ice for 20 minutes and the aggregates were spun down for bulk removal of unwanted proteins. Thereafter, (HE)_3_-Z_HER3_ was purified on an ÄKTAexplorer (GE Healthcare, Uppsala, Sweden) using a 3 ml Ni Sepharose 6 Fast Flow column (GE Healthcare). Finally, the buffer of the eluate was changed to 20 mM NH_4_Ac (pH 5.5) and the proteins were freeze-dried.

(HE)_3_-Z_HER3_ was dissolved in 20 mM NH_4_Ac (pH 5.5) and reduced with a molar concentration of tris(2-carboxyethyl)phosphine (TCEP) equal to the protein concentration for 30 min at 37 °C. The proteins were incubated at 37 °C for 90 min with a ten-fold molar excess of maleimide derivatives of DOTA and DOTAGA (CheMatech) for site-specific conjugation to a C-terminal cysteine on the affibody. Metal ion contaminations were removed from all buffers used with Chelex 100 resin (Bio-Rad Laboratories).

For purification, reverse-phase high performance liquid chromatography (RP-HPLC) on a 1200 series HPLC system using a Zorbax 300SB-C18 semi-preparative column (Agilent Technologies, Santa Clara, CA) was used. Water with 0.1% trifluoroacetic acid was used as running buffer and an acetonitrile gradient was used for elution.

The molecular mass of the conjugates was confirmed with electrospray ionization mass spectrometry (ESI-MS) using a 6520 Accurate-Mass Q-TOF LC/MS (Agilent Technologies).

### Characterization

Characterization of affibody conjugates was done as previously described^40^.

The purity of the conjugates was determined with RP-HPLC using an analytical Zorbax 300SB-C18 column (Agilent Technologies).

Alpha-helical content, thermal stability and refolding capacity of all conjugates were analyzed by circular dichroism spectroscopy (Chirascan spectropolarimeter Applied Photophysics, United Kingdom) with an optical path length of 1 mm at a concentration of 0.25 mg/ml.

The thermal stability was evaluated by measuring the change in ellipticity at 221 nm during heating (5 °C/min) from 20 to 90 °C. The melting temperatures (T_m_) were estimated from the data acquired from variable temperature measurements (VTM) by curve fitting using a Boltzmann Sigmoidal model (GraphPad Prism, version 7). Spectra obtained from measurements at wavelengths in the range 195–260 nm at 20 °C, before and after thermal denaturation, were used to study the refolding capacity of the conjugates.

Binding affinity towards human HER3 was investigated using surface plasmon resonance (SPR) on a Biacore T200 system (GE Healthcare). The analysis was performed using single-cycle kinetics on a CM5 sensor chip with immobilized human HER3-Fc (Sino Biological). Five concentrations (3.125, 6.25, 12.5, 25 and 50 nM) of each conjugate were sequentially injected in a single cycle with a contact time of 150 seconds for each concentration.

### Labeling and stability

^68^Ga/^68^Ge-generator (Cyclotron Co. Obninsk, Russia) was eluted over 5–6 minutes with 0.1 M HCl (800 µl/min) in fractions of 400 ul. The third fraction was used for radiolabeling. (HE)_3_-Z_HER3_-NODAGA was labeled identically to the protocol described before^40^. (HE)_3_-Z_HER3_-DOTA and (HE)_3_-Z_HER3_-DOTAGA were labeled as follows. 20 µg of (HE)_3_-Z_HER3_-X (X = DOTA, DOTAGA) were buffered in 300 µl sodium acetate (1.25 M, pH 3.6) and incubated with 100–150 MBq gallium-68 eluate for 15 minutes at 85 °C. Labeling yields were analyzed with instant thin layer chromatography (ITLC). After labeling [^68^Ga]Ga-(HE)_3_-Z_HER3_-X was incubated with a 1000-fold molar excess of EDTA for 10 minutes at 85 °C to remove loosely bound gallium-68 from the affibody molecules. Distribution of activity was thereafter analyzed with ITLC. To achieve purity > 98% [^68^Ga]Ga-(HE)_3_-Z_HER3_-X was separated from the labeling mixture using NAP5-size exclusion columns. Purity was then analyzed using ITLC.

To test stability, 1 µg of [^68^Ga]Ga-(HE)_3_-Z_HER3_-X was incubated for 1 hour in PBS at room temperature or in human serum at 37 °C. After incubation, the activity distribution was measured by ITLC.

### *In vitro* analysis of [^68^Ga]Ga-(HE)_3_-Z_HER3_-DOTA or [^68^Ga]Ga-(HE)_3_-Z_HER3_-DOTAGA

All cell experiments were performed in triplicates on HER3-expressing cell lines BxPC-3 and DU145. Cells were plated in 35 mm dishes 1 day before the experiments. Assays were performed according to previously described protocols^27,40^.

*In vitro* characterization of [^68^Ga]Ga-(HE)_3_-Z_HER3_-NODAGA was published earlier by Dahlsson *et al*. 2019^40^.

To test for specific binding of [^68^Ga]Ga-(HE)_3_-Z_HER3_-DOTA and [^68^Ga]Ga-(HE)_3_-Z_HER3_-DOTAGA towards HER3, HER3 receptors were blocked by addition of 50 nM unlabeled Z_HER3_. After 10 minutes incubation at room temperature, 0.1 nM of [^68^Ga]Ga-(HE)_3_-Z_HER3_-DOTA or [^68^Ga]Ga-(HE)_3_-Z_HER3_-DOTAGA was added and samples were incubated for 1 hour at 37 °C. Thereafter, the cell samples were measured for activity content in the automated gamma counter.

To study the internalization of the compounds, BxPC-3 and DU145 cells were continuously incubated with 0.1 nM of either [^68^Ga]Ga-(HE)_3_-Z_HER3_-DOTA or [^68^Ga]Ga-(HE)_3_-Z_HER3_-DOTAGA for up to 4 hours. At selected time points, the membrane-bound activity fraction was collected after 5 minutes incubation with 0.2 M glycine buffer (0.15M NaCl, 4 M Urea, pH 2) on ice. The remaining activity was considered internalized and collected after incubating the cells with 1 M NaOH for 30 minutes at 37 °C.

### *In vivo* experiments

*In vivo* experiments were carried out as described previously^40^ in compliance with national legislation on protection of laboratory animals and permission from the Ethics Committee for Animal Research in Uppsala, Sweden (approval number C5/16 from 26-02-2016).

Female Balc/c nu/nu mice were implanted subcutaneously with 5 × 10^6^ cells/animal 20 days prior to the experiment. At the time of the experiment, the average tumor weight was 0.06 ± 0.03 g. Average mouse weight was 19 ± 1 g.

Mice were injected with 2 µg (0.7 MBq, 100 µl) of [^68^Ga]Ga-(HE)_3_-Z_HER3_-DOTA and [^68^Ga]Ga-(HE)_3_-Z_HER3_-DOTAGA. Previously studied [^68^Ga]Ga-(HE)_3_-Z_HER3_-NODAGA^40^ was included for reference.

Mice were pre-injected with Ketalar-Rompun solution 10 mg/mL Ketalar and 1 mg/mL Rompun; 20 µL solution/gram of body weight) and sacrificed 3 h pi. Tumors and samples from blood, mErbB3 expressing organs (salivary gland, lung, liver, stomach, small intestine), spleen, kidney muscle and bone were collected. Samples were weighed and measured for activity content in the automated gamma counter. Uptake is presented as %ID/g. GI and carcass were collected and activity was measured. Uptake was presented as %ID.

To confirm binding specificity of [^68^Ga]Ga-(HE)_3_-Z_HER3_-DOTA and [^68^Ga]Ga-(HE)_3_-Z_HER3_-DOTAGA the injected protein dose was adjusted to 70 µg. A biodistribution experiment was done according to the protocol described above.

## Supplementary information

Supplementary materials and results
