# Supplementary material for: Increase in negative charge of 68Ga/chelator complex reduces unspecific hepatic uptake but does not improve imaging properties of HER3-targeting affibody molecules
Source: Sci Rep. 2019 Nov 27;9:17710. doi: 10.1038/s41598-019-54149-3 (PMC6881397; doi:10.1038/s41598-019-54149-3)
Supplement: Supplementary file 1 — Supplementary materials and results [file 41598_2019_54149_MOESM1_ESM.docx]

***Supplementary Material***

**Increase in negative charge of ^68^Ga/chelator complex reduces unspecific hepatic uptake but does not improve imaging properties of HER3-targeting affibody molecules**

**Sara S. Rinne^†^, Charles Dahlsson Leitao, Joshua Gentry, Bogdan Mitran, Ayman Abouzayed, Vladimir Tolmachev, Stefan Ståhl, John Löfblom, and Anna Orlova**

**Material and Methods**

*Production, conjugation and purification*

Affibody molecules (HE)_3_-Z_HER3:08698_-NODAGA, (HE)_3_-Z_HER3:08698_-DOTA and (HE)_3_-Z_HER3:08698_-DOTAGA were produced, purified and characterized as previously described (Dahlsson et al, 2019).

The HER3-binding affibody (HE)_3_-Z_HER3:08698_ (further denoted as (HE)_3_-Z_HER3_) was produced in BL21*(DE3) *E. coli* (Thermo Fisher Scientific) in an overnight culture at 25°C after induced expression with 100 μM IPTG at an OD_600_ of 0.8.

Cells were lysed with French press and the supernatant was heated to 90°C for 10 min followed by incubation on ice for 20 minutes and the aggregates were spun down for bulk removal of unwanted proteins. Thereafter, (HE)_3_-Z_HER3_ was purified on an ÄKTAexplorer (GE Healthcare, Uppsala, Sweden) using a 3 ml Ni Sepharose 6 Fast Flow column (GE Healthcare). Finally, the buffer of the eluate was changed to 20 mM NH_4_Ac (pH 5.5) and the proteins were freeze-dried.

(HE)_3_-Z_HER3_ was dissolved in 20 mM NH_4_Ac (pH 5.5) and reduced with a molar concentration of tris(2-carboxyethyl)phosphine (TCEP) equal to the protein concentration for 30 min at 37°C. The proteins were incubated at 37°C for 90 min with ten-fold molar excess of maleimide derivatives of NODAGA, DOTA and DOTAGA (CheMatech) for site-specific conjugation to a C-terminal cysteine on the affibody. Metal ion contaminations were removed from all buffers used with Chelex 100 resin (Bio-Rad Laboratories).

For purification, reverse-phase high performance liquid chromatography (RP-HPLC) on a 1200 series HPLC system using a Zorbax 300SB-C18 semi-preparative column (Agilent Technologies, Santa Clara, CA) was used. Water with 0.1% trifluoroacetic acid was used as running buffer and an acetonitrile gradient was used for elution. The acetonitrile gradient used for purification was 25-31% over 20 min, 27-33% over 15 min and 25-35% over 15 min for DOTA-, DOTAGA- and NODAGA-conjugates respectively. Representative chromatograms from RP-HPLC purification are shown in Figure S7.

Molecular mass of the conjugates was confirmed with electrospray ionization mass spectrometry (ESI-MS) using a 6520 Accurate-Mass Q-TOF LC/MS (Agilent Technologies). For separation, a C4 PepMap300, 5 um, 300 Å (Thermo Scientific) column was used with a 5-95% gradient over 30 seconds of acetonitrile + 0.1% formic acid. For acquisition, a mass range of 300-3000 m/z, a spectra rate of 1 Hz and positive ion polarity were used

*Characterization*

The purity of the conjugates was determined with RP-HPLC using an analytical Zorbax 300SB-C18 column (Agilent Technologies) and an acetonitrile gradient of 20-50% over 20 min.

Alpha-helical content, thermal stability and refolding capacity of all conjugates were analyzed by circular dichroism spectroscopy (Chirascan spectropolarimeter Applied Photophysics, United Kingdom) with an optical path length of 1 mm at a concentration of 0.25 mg/ml.

The thermal stability was evaluated by measuring the change in ellipticity at 221 nm during heating (5°C/min) from 20 to 90°C. The melting temperatures (T_m_) were estimated from the data acquired from variable temperature measurements (VTM) by curve fitting using a Boltzmann Sigmoidal model (GraphPad Prism, version 7). Spectra obtained from measurements at wavelengths in the range 195-260 nm at 2 °C, before and after thermal denaturation, were used to study the refolding capacity of the conjugates.

Binding affinity towards human HER3 was investigated using surface plasmon resonance (SPR) on a Biacore T200 system (GE Healthcare). The analysis was performed using single-cycle kinetics on a CM5 sensor chip with immobilized human HER3-Fc (Sino Biological). Five concentrations (3.125, 6.25, 12.5, 25 and 50 nM) of each conjugate were sequentially injected in a single cycle with a contact time of 150 seconds for each concentration.

**Results**

The (HE)_3_-tagged HER3-binding affibody (HE)_3_-Z_HER3_ was purified with IMAC, followed by coupling to maleimide derivatives of DOTA, DOTAGA and NODAGA. Structural overview of the compounds is presented in Figure S1. The proteins were thereafter subjected to RP-HPLC purification in order to remove remaining chelator and for separation from unconjugated protein. The purity, determined with RP-HPLC, exceeded 95% for all conjugates (Fig. S2). The experimental molecular mass of each conjugate was in perfect agreement with the theoretical mass, as determined by ESI-MS (Fig. S3). Notably, the mass determination revealed non-processed N-terminal methionine for all conjugates, due to the presence of the (HE)_3_-tag at the N-terminus.

The alpha-helical content, thermal stability and refolding of the conjugates were investigated with circular dichroism spectroscopy. Thermal denaturation curves are shown in Fig. S4 and the associated melting temperatures are presented in table S1. Complete refolding was observed for each conjugate following thermal denaturation by comparison of spectra obtained at 20°C before and after denaturation. Kinetic data acquired from SPR analysis are presented in Table S1 as the average from duplicate injections. K_D_ values refer to the monovalent affinity for human HER3 according to a Langmuir 1:1 model. Representative sensorgrams with fitted curves for each conjugate are shown in Fig S5.


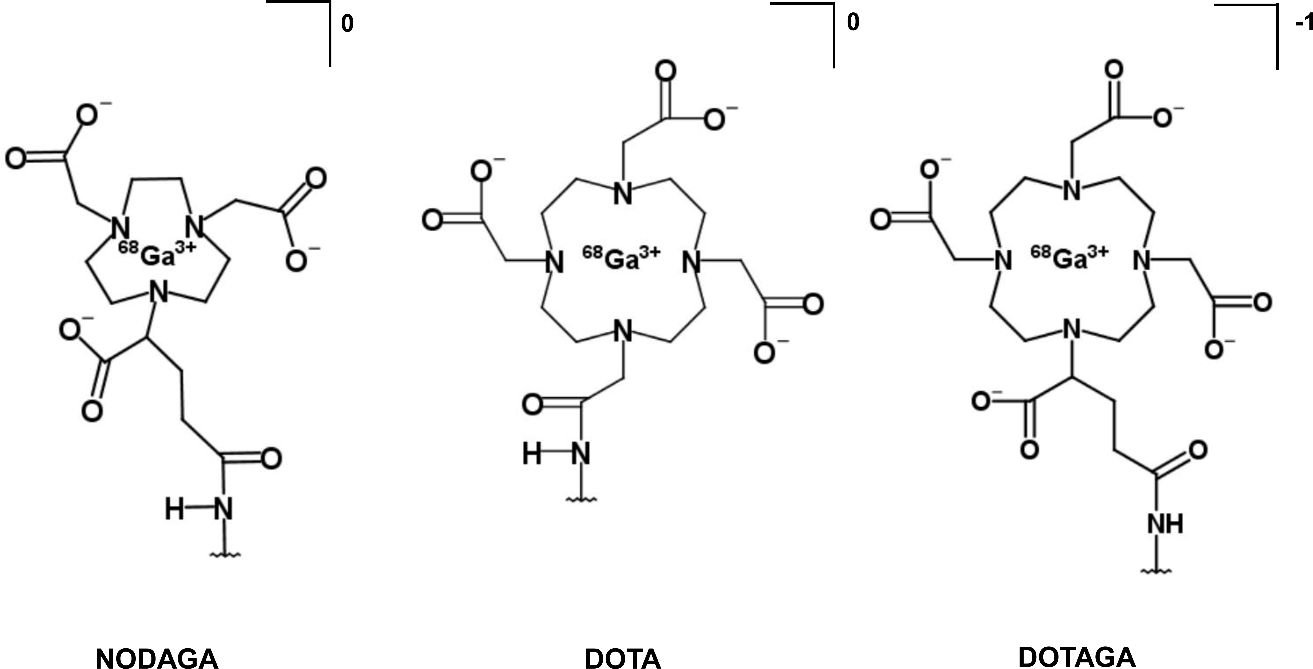


**Figure S1**. Structural overview over the tested ^68^Ga-chelator complexes conjugates to the C-terminus of (HE)_3_-Z_HER3_ via a C-terminal cysteine.

**Table 1:** Melting temperatures (T_m_) of the conjugates. *Data for [^68^Ga]Ga-(HE)_3_-Z_HER3_-NODAGA was previously reported by [40].

| **Conjugate** | **T_m_ (°C)** |
| --- | --- |
| (HE)_3_-Z_HER3_-NODAGA* | 65.0 |
| (HE)_3_-Z_HER3_-DOTA | 65.3 |
| (HE)_3_-Z_HER3_-DOTAGA | 65.1 |


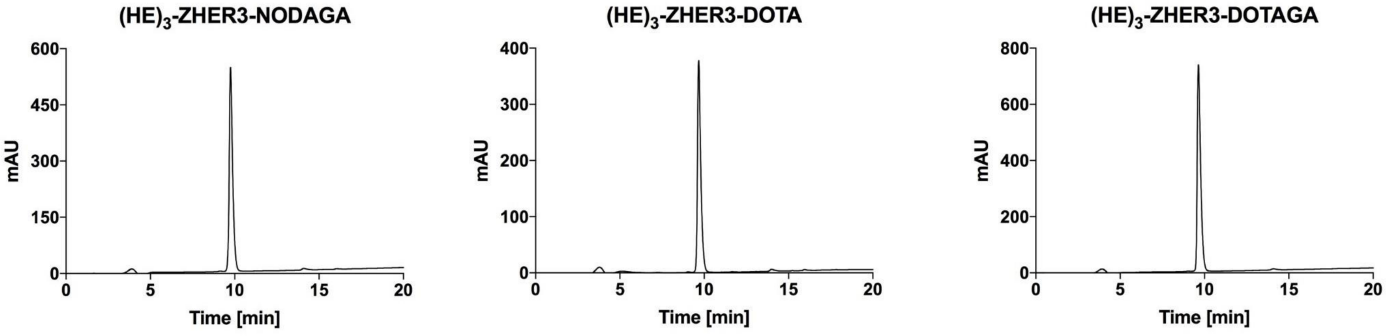


**Figure S2 Purity**. Chromatograms from analysis using RP-HPLC with absorbance at 220 nm on the y-axis. Results for (HE)_3_-Z_HER3_-NODAGA were published previously by [40].


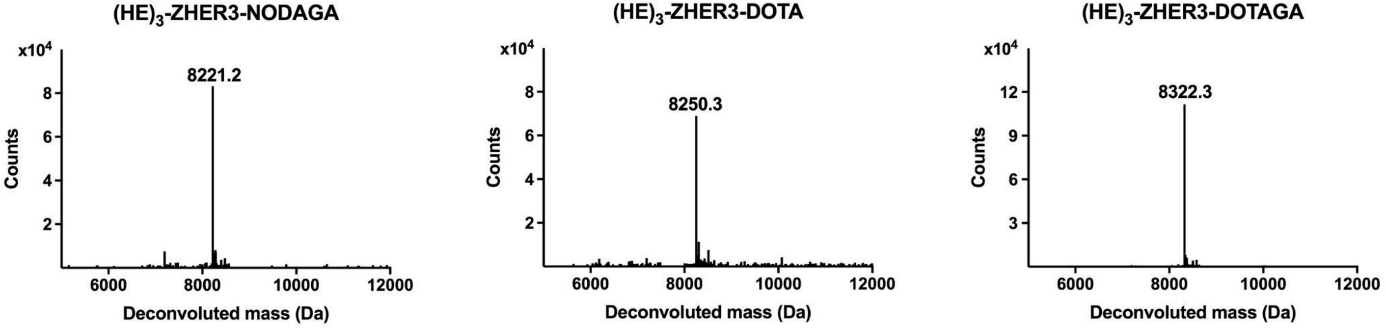


**Figure S3: Mass Determination.** Mass spectra from ESI-MS with the experimental molecular weight for the main peaks indicated. Results for (HE)_3_-Z_HER3_-NODAGA were published previously by [40].


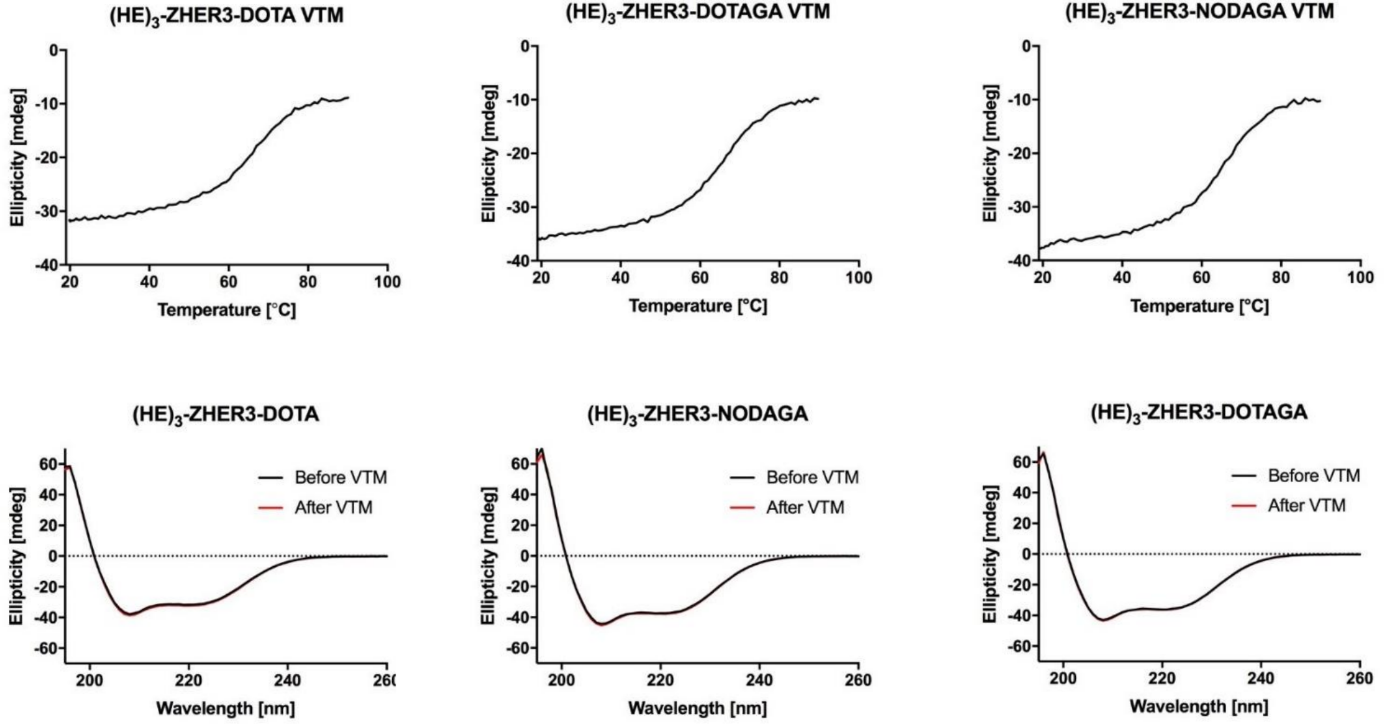


**Figure S4: Thermal stability (top) and refolding capacity (bottom).** Top: Thermal stability was evaluated, using variable temperature measurement (VTM), by observing the change in ellipticity at 221 nm while heating the sample from 20°C to 90°C. Bottom: Superimposed circular dichroism spectra, measured at 20°C and in the range 195-260 nm, before and after thermal denaturation. Results for (HE)_3_-Z_HER3_-NODAGA were published previously by [40].


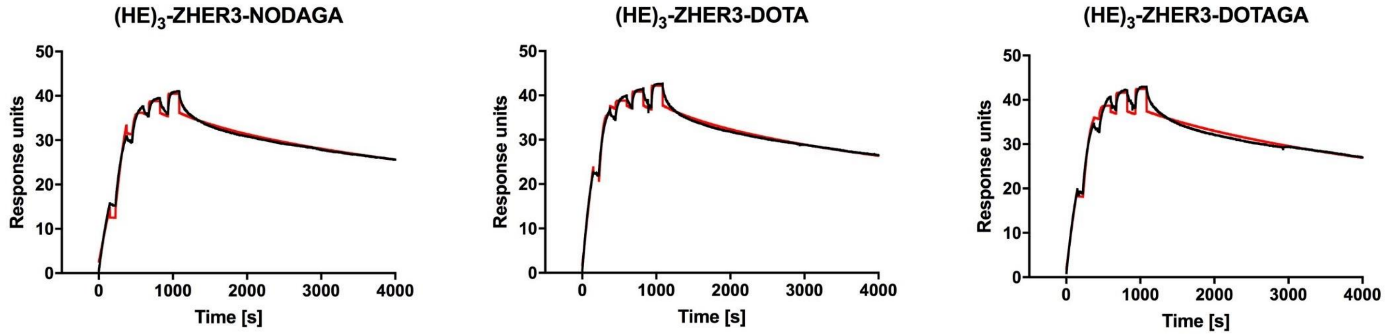


**Figure S5: SPR analysis.** Representative experimental sensorgrams (black) with fitted curves (red). Immobilized human HER3 was subjected to five concentrations (3.125, 6.25, 12.5, 25 and 50 nM) of NODAGA-, DOTA- and DOTAGA-conjugated affibody. Results for (HE)_3_-Z_HER3_-NODAGA were published previously by [40].


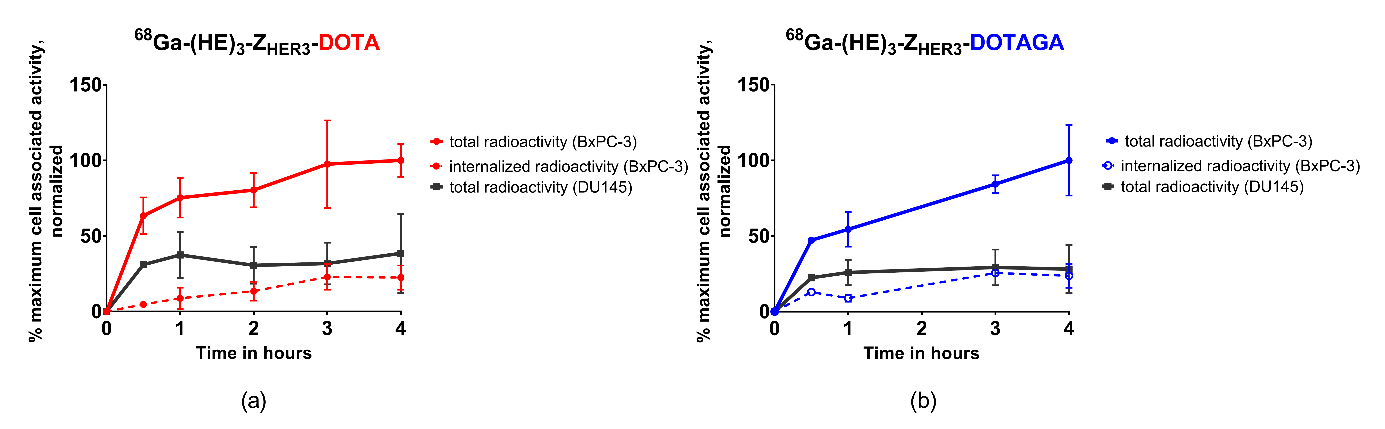


**Figure S6:** **Cellular processing** on BxPC-3 cells were continuously incubated with 0.1 nM of (a) [^68^Ga]Ga-(HE)_3_-Z_HER3_-DOTA or (b) [^68^Ga]Ga-(HE)_3_-Z_HER3_-DOTAGA for 4 hours. Experiments were performed on both cell lines in parallel using the same stock solution of the radiolabeled affibody molecules. Cellular processing of [^68^Ga]Ga-(HE)_3_-Z_HER3_-NODAGA was previously described [40].
